# Coexistence of Primary and Secondary Delusional Parasitosis

**DOI:** 10.1155/2020/2537014

**Published:** 2020-07-28

**Authors:** Kourtne Roberts, Aparna Das, Michael Fuller, Christopher Thomas

**Affiliations:** Department of Psychiatry, University of Texas Medical Branch, Galveston, Texas 77555, USA

## Abstract

**Objective:**

Delusional parasitosis (DP) is a difficult-to-treat condition. It is prevalent in all cultures, societies, and countries. Previous case reports of DP have been published; however, the patient presented in this report is unique because of the co-occurrence of both primary and secondary types of DP. We aim to explore the two subtypes of DP.

**Conclusions:**

We discuss DP from a nosological perspective and also highlight the various theories at play in the pathogenesis of primary and secondary DP. The different subtypes of DP should be taken into consideration as they also have a bearing on the management.

## 1. Introduction

Delusional parasitosis (DP) is a false, firm, unshakable belief that one is infested with bugs, parasites, worms, mites, lice, insects, or similar creatures, held with delusional intensity [1]. DP was mentioned as early as 17th century in France [2]. The clinical picture of DP was reported by Robert Willam in 1799, Johann Heinrich Jὂrdens in 1801, and Charcellay in 1843. It is widely accepted that Thiebierge (1894) and Perrin (1896) were the first authors to fully describe DP [1, 2]. Over the years, DP has been referred to as “delusory parasitosis, delusions of parasitosis, delusion of infestation, dermatozoenwahn (dermatozoic delusion) [3], insect hallucinations, pseudoparasitism, and hallucinations of insect” [4]. It is also popularly known as Ekbom syndrome after the Swedish neurologist Karl Axel Ekbom who published seminal accounts of the disease in 1937 and 1938 [2, 5].

DP has a wide spectrum of delusional subtypes and multiple specifiers and related terms [2–6]. It can still be considered an under-explored territory as not many epidemiological studies have been conducted. Although its exact prevalence and incidence are not known with certainty, some parameters have shown a reliable trend. For example, in Trabert's meta-analysis of 1223 cases of DP, it was found that the mean age of these patients was 57 years, and the ratio of female to male patients was 1.4 : 1 for persons aged <50 years and 2.5 : 1 for those aged >50 years [7]. Likewise, most studies have found that DP is a disorder of the middle-aged and elderly and more women suffer from DP as compared to men [4].Patients come from all socioeconomic classes; some are even physicians [8]. They are often reported to be socially isolated, but it is not documented whether the isolation is the result of the delusions or is a contributory factor [4, 7].

The clinical course of DP is variable and can be episodic, acute, or chronic. Primary and secondary DP both have a chronic course, although secondary DP due to intoxication may have an acute course. An episodic state is generally seen with episodic illnesses like recurrent depressive disorder [9]. According to Trabert, the mean duration of the delusion was 3.0 ± 4.6 years (median: 1 year) [7]. The course of DP is not as unfavorable as commonly thought; in about half the patients, a full remission was described during the observation period [7]. Short preclinical courses may indicate a better outcome [7].

## 2. Case

A 49-year-old Caucasian male with no past psychiatric treatment was admitted for management of congestive heart failure. He was seen by the consultation and liaison psychiatry team for psychiatric clearance prior to placement of a left ventricular assist device. During evaluation, the patient was found to have parasitic delusions.

Patient reported that around 9 months prior to evaluation, he began experiencing “sharp, burning, shooting pains” in both lower extremities. He attributed this to a “boring insect.” Shortly after this, he and his wife separated, resulting in him moving out of their home. Thereafter, he began to feel insects entering his nose and left ear.

He sprayed his residence repeatedly using professional-grade extermination chemicals and took daily bleach baths to treat the believed infestation. He stated that he saw the insects leave his body while bathing and collected samples of them. He set up cameras in his home to monitor the insects' activities. He had scars on his legs from attempting to dig the insects out but did not find this effective for their removal.

The patient described himself as a “clean freak and perfectionist.” He worked as an exterminator for more than 10 years. He had been taking hydroxyzine 25 mg nightly dose as needed for at least 25 years for vague pruritus for which no cause had ever been found. The patient had a past medical history of diabetes mellitus (DM) that was not very controlled with metformin and insulin. While hospitalized, the patient underwent extensive evaluation and all basic lab investigations were normal. The team ruled out other causes like schizophrenia, psychotic depression, drug-induced psychosis, formication without delusion, withdrawal from cocaine, amphetamines or alcohol, vitamin B12 deficiency, multiple sclerosis, cerebrovascular disease, syphilis, and dermatologic infection.

Patient was initially diagnosed with secondary DP due to diabetic neuropathy. He was started on gabapentin, titrated to 300 mg twice a day which resolved his lower extremity neuropathic pain. This also relieved his delusions of parasitic infestation of his legs. However, he continued to believe his nose and left ear contained insects. His delusions related to infestation of nose and ears were considered to be primary as diabetic neuropathy is not typically seen over the face. He was started on olanzapine 2.5 mg at bedtime for treatment of these delusions. There was no improvement in his delusion. Olanzapine uptitration was discussed, but patient refused to try a higher dose. The patient was rejected from having the proposed procedure due to inability to comply with fluid restrictions and smoking cessation. Despite aggressive management of his medical condition, his cardiovascular status continued to decompensate, and he elected to go home on hospice.

## 3. Discussion

The patient's DP can be classified as both primary and secondary DP over the course of his illness. The authors are unaware of any other similar report in the literature. A review of the currently known information about pathophysiology of DP provides some insight on these issues. There are four separate studies on the heritability of DP that concluded patients with DP have a higher genetic susceptibility to mental illness compared to controls, based both on a positive family history of both DP and other psychiatric conditions [4]. Neuronal aberrations and neuroanatomical changes documented in patients with DP include diffuse atrophy in cortical and/or subcortical areas, areas of infarction and degeneration in the basal ganglia, striatum, and putamen, poor blood flow to frontotemporal and temporal-parietal areas, and lesions in the above-named structures [4].

The leading argument in the neuropathogenesis of DP concerns aberrations in dopamine neurotransporters. Huber et al. hypothesized decreased striatal dopamine transporter (DAT) functioning, with an increased extracellular dopamine level, as the etiologic condition for DP, both primary and secondary [10]. DAT is a presynaptic plasma membrane protein densely represented in the striatum. It is known that DAT inhibitors, like amphetamine derivatives, which block synaptic reuptake of dopamine, can induce the clinical expression of DP-like symptoms [4]. The increased incidence of primary DP associated with aging is hypothetically explainable due to an age-related decline of DAT density, likely genetically caused via decreased DAT1-gene expression. Causes of secondary DP may cause decreased striatal DAT functioning, via various mechanisms including blocking, reduced ligand binding, reduced density, and reduced activity [10].

The proposed role of dopaminergic transmission in the biological basis of DP is supported by the role of dopaminergic-blocking agents in the treatment of DP. Although there are no controlled clinical trials, systematic reviews have shown evidence that high-potency typical antipsychotics like haloperidol and pimozide [1], and atypical antipsychotics including risperidone, olanzapine, and amisulpiride may be effective in DP [11, 12].

Coltheart et al. proposed the “two factor model” for the development of all monothematic delusions, including DP which puts the syndrome into more practical terms. This theory explains that patients with DP have two “factors,” or abnormalities, leading to the formation of the patient's delusion. The first factor is the “impetus” and is different for each patient [13]. In the case detailed above, the first factor was abnormal sensation. The second factor (thought to be common to all delusional disorders) is dysfunction of the right frontal cortex that leads to “a dysfunctional belief evaluation system” and prevents the person from “rejecting the belief in the light of strong evidence against it” [13]. They explain that both factors are required for the development of a monothematic delusion. For example, a patient with right frontal dysfunction would not develop a delusion unless there was an abnormal sensory input and vice versa. This would explain how a patient, such as the one described, could have primary, secondary, or both presentations of delusions. The abnormal sensation created by neuropathic pain in the patient's legs, when combined with frontal cortex dysfunction likely led to symptoms that would be categorized as secondary. These symptoms resolved with treatment of his neuropathic pain, which was the first factor in the model. However, no secondary or functional cause could be found for the symptoms of DP involving his nose and mouth. Thus, these symptoms would be categorized as primary.

Regardless of etiology, there are reasons to consider primary and secondary DP to be separate processes, such as course, prognosis, and treatment. A case-based analysis by Freudenmann et al. showed that 75% of patients responded to atypical antipsychotics, 38% had partial, and 37% had full remission of DP [11]. When comparing primary to secondary response rates, patients with secondary DP had a partial or full response rate of 80% versus those with primary DP who had a response rate of 68% [11]. In cases of secondary DP, treatment of the underlying illness in conjunction with an antipsychotic is indicated, versus treatment of primary DP is antipsychotics as monotherapy [11]. For example, in the case of the patient listed above, the patient presented with burning pain in lower extremities which the patient attributed to “boring insects.” He had the typical presentation of peripheral neuropathy likely related to his long standing DM. Treatment with gabapentin resolved this pain and specific aspect of his secondary DP but his primary delusion continued to persist.

## 4. Conclusion

DP is a condition frequently seen by psychiatrists, dermatologists, and primary care physicians. It is imperative to do a thorough evaluation as patients could have primary delusion or secondary subtype, one that is comorbid with other psychiatric and/or medical conditions. Treatment of comorbid psychiatric and medical conditions may ameliorate some symptoms of secondary DP. Our case illustrates that co-occurrence of primary and secondary forms could also be a possibility that should be kept in mind. Awareness of such a coexistence may lead to better management strategies and hence better overall course and prognosis of the illness.
